# Submicron Plastic Adsorption by Peat, Accumulation in Sphagnum Mosses and Influence on Bacterial Communities in Peatland Ecosystems

**DOI:** 10.1021/acs.est.2c04892

**Published:** 2022-11-03

**Authors:** Mandar Bandekar, Fazel Abdolahpur Monikh, Jukka Kekäläinen, Teemu Tahvanainen, Raine Kortet, Peng Zhang, Zhiling Guo, Jarkko Akkanen, Jari T. T. Leskinen, Miguel A. Gomez-Gonzalez, Gopala Krishna Darbha, Hans-Peter Grossart, Eugenia Valsami-Jones, Jussi V. K. Kukkonen

**Affiliations:** † Department of Environmental and Biological Sciences, University of Eastern Finland, Joensuu-Kuopio 80101, Finland; ‡ Department of Plankton and Microbial Ecology, Leibniz Institute for Freshwater Ecology and Inland Fisheries, 16775 Stechlin, Germany; § School of Geography, Earth and Environmental Sciences, University of Birmingham, Edgbaston, Birmingham B15 2TT, U.K.; ∥ SIB Labs, University of Eastern Finland, 70211 Kuopio, Finland; ⊥ Diamond Light Source, Harwell Science and Innovation Campus, Didcot, Oxfordshire OX11 0DE, U.K.; # Environmental Nanoscience Laboratory, Department of Earth Sciences, Indian Institute of Science Education and Research Kolkata, Mohanpur, West Bengal 741246, India; ○ Institute of Biochemistry and Biology, Potsdam University, 14469 Potsdam, Germany

**Keywords:** mesocosm, Sphagnum moss, poly(vinyl chloride), polystyrene, gadolinium entrapped particles, accumulation

## Abstract

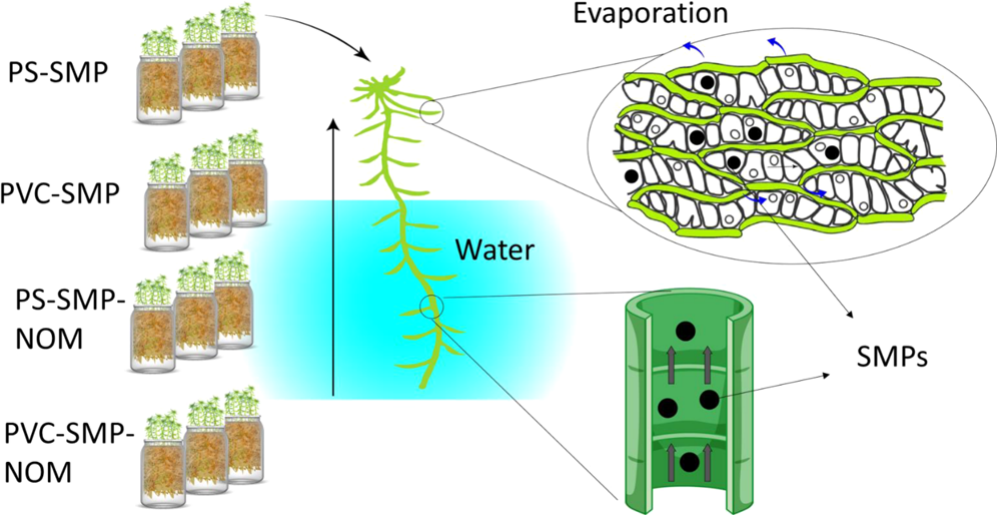

The smallest fraction of plastic pollution, submicron plastics (SMPs <1 μm) are expected to be ubiquitous in the environment. No information is available about SMPs in peatlands, which have a key role in sequestering carbon in terrestrial ecosystems. It is unknown how these plastic particles might behave and interact with (micro)organisms in these ecosystems. Here, we show that the chemical composition of polystyrene (PS) and poly(vinyl chloride) (PVC)-SMPs influenced their adsorption to peat. Consequently, this influenced the accumualtion of SMPs by *Sphagnum* moss and the composition and diversity of the microbial communities in peatland. Natural organic matter (NOM), which adsorbs from the surrounding water to the surface of SMPs, decreased the adsorption of the particles to peat and their accumulation by *Sphagnum* moss. However, the presence of NOM on SMPs significantly altered the bacterial community structure compared to SMPs without NOM. Our findings show that peatland ecosystems can potentially adsorb plastic particles. This can not only impact mosses themselves but also change the local microbial communities.

## Abstract 2

This study shows that submicron plastics adsorb to peat in peatland ecosystems and have adverse effects on plant and bacteria.

## Introduction

Peatlands are terrestrial wetland ecosystems, where the production of natural organic matter (NOM) by plants such as *Sphagnum* moss exceeds its decomposition by microorganisms resulting in the net accumulation of peat.^1^ The process of peat formation has resulted in the accumulation of approximately one-third of world soil carbon in peatlands, although they cover only 3% of the earth’s surface.^2,3^ This indicates the immense importance of these ecosystems in storing the carbon absorbed by plants from the atmosphere within peat soils. Therefore, the balance between production by plants and consumption by bacteria in peatlands is critical on the global scale.^4^ Anthropogenic disturbances, such as pollution, fire, and peat extraction,^5^ have been reported to substantially affect peatland ecosystems,^6^ which make these ecosystems the most threatened habitats in Europe.^7^ These change major ecosystems’ properties, i.e., transform peatlands from sinks to sources of carbon dioxide (CO_2_).^5^

The physicochemical properties make peat an effective adsorbent for various anthropogenic chemicals and materials.^8^ Accumulation of partially decomposed plants in peatlands forms compact porous structures^9^ with high polarity and large surface area.^10^ Peat also has a high water holding capacity, typically containing 80–90% water of fresh weight in natural peatlands.^10^ For example, it has been documented that peats have a strong adsorption affinity for oil^11^ and heavy metals such as zinc, lead, and mercury.^12^ Plastic particles are anthropogenic materials that can potentially adsorb and retain in peat. Since an increasing number of studies are reporting the ubiquitous presence of microplastics (1 μm < particle size < 5 mm) and submicron plastic (SMPs: particle size < 1 μm) in different environmental compartments, from mountain^13^ to deep sea^14^ and from tropical regions to polar areas,^15,16^ it is likely that also peatland ecosystems are contaminated with these anthropogenic materials. Plastic particles may be transferred to peatland by runoff, effluent discharge, and atmospheric deposition, as reported for other ecosystems.^17−19^ Plastic particles in nature represent a wide variety of polymer types, including, e.g., polystyrene (PS), polyethylene (PE), and poly(vinyl chloride) (PVC). In general, plastics have hydrophobic surfaces and the degree of hydrophobicity depends on the type (chemical composition) of the plastics (e.g., PVC < PS < PE).^20^ One expects that when SMPs enter the porous structures of peat, the hydrophobic surfaces and the large surface area-to-volume ratio of the particles increase their tendency to adsorb to the surfaces of peats. As a result, peatlands might act as an important sink for SMPs.^21^ Very limited information is available about the presence of microplastics in peatlands and their effects on the peatland ecosystem,^21,22^ and particularly, no information is available on the possible impact of SMPs in peatlands.

The high free surface energy of SMPs allows the particles to adsorb NOM from the surrounding water on their surfaces.^23^ NOM is a mixture of organic compounds with different molecular sizes, structures, and functional groups, which originates from the natural decomposition of plants and organisms in water. The NOM layer on SMPs can act as the interface of the particles, thus influencing the behavior, fate, and interaction of the particles with surrounding surfaces. Since NOM change the particle surface charge, it influences colloidal stability and particle behavior.^24^ For example, the presence of NOM on the surface of SMPs may alter the sorption of the particles to the peat’s surfaces due to steric stabilization.^23^ Lessons learned from nanomaterials fate assessment studies show that NOM on the surface of copper and PS nanoparticles can increase their dispersion stability in freshwater ecosystems.^25,26^ This is critical for risk assessment of SMPs in peatland ecosystems because changes in the dispersion stability of SMPs can alter the bioavailability of the particles to plants and consequently their impact on (micro)organisms.

The microbiomes of peatlands, which greatly differ from the generally studied bacterial model systems, are of paramount importance for the ecosystem. Because they not only directly control the turnover of organic carbon in peatland^27^ but also play profound roles in carbon, nitrogen, phosphorus, sulfur, and metal biogeochemical cycles in peatland ecosystem as well as vegetation community structure, productivity,^27,28^ and plant defense.^28^ Though the microbial composition of pristine peatlands is considered relatively stable,^29^ evidence^30,31^ shows that changing environmental conditions can affect the biological diversity of microbial communities. Despite the important role of the microbiome in the peatland ecosystem functioning, no information is available on the influence of SMPs on the microbial community structure and function in peatlands.

**Figure 1:**
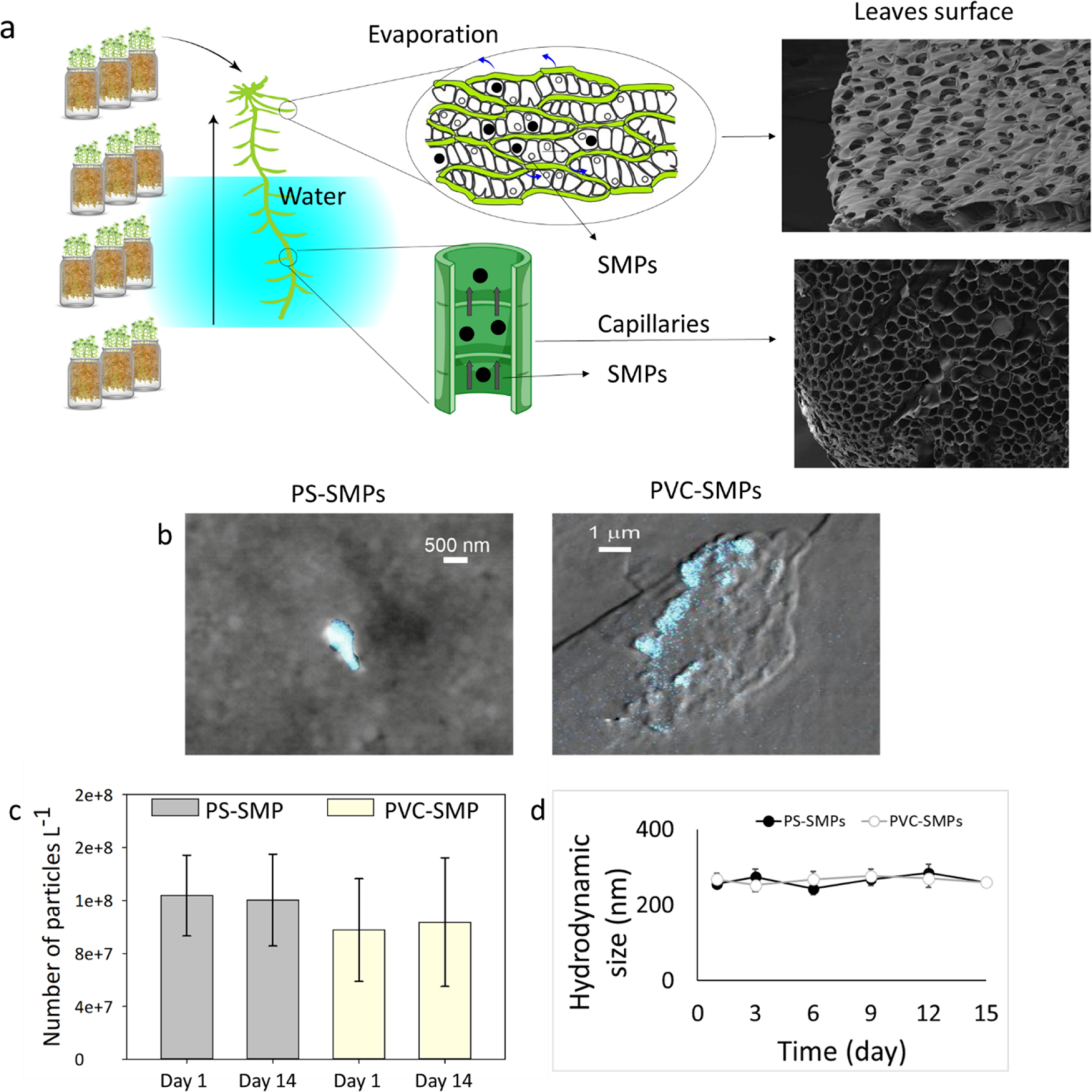
(a) Schematic representation of the expected SMPs uptake by moss and their transportation in the shoots as a result of particle diffusion facilitated by capillary forces. The surface of the leaves and the shoots were imaged using SEM to show the pores on the surfaces and the capillaries in the shoots. (b) Differential phase contrast (DPC) gradient phase of the PS-SMPs and PVC-SMPs in grayscale with the Gd signal in blue measured using X-ray fluorescence, showing the presence of Gd (blue) in the particle agglomerates. (b) Number of SMP particles measured using spICP-MS on days 1 and 14 after incubation in distilled water. (c) Hydrodynamic size of the SMP particles measured over time to show the stability of the particles against agglomeration.

The uptake and accumulation of SMPs by plants can be influenced by the plant species and the physicochemical properties of the particles such as size and chemical composition. Recently, it was documented that Gymnosperm plants such as lettuce (*Lactuca sativa*) can take up PS-SMPs by root from water and accumulate these particles in their tissues.^28,32^ Water transport mechanisms in bryophytes, like the *Sphagnum* mosses, are fundamentally different from those of gymnosperm because mosses have neither roots nor leaf stomata.^33^ Leaves grow on the stem and the branches and the lowermost parts of the plant form peat.^34,35^ Water diffusion in mosses occurs across the plant surface, which has a simple structure that is often just one cell thick, and the movement of the water is facilitated by capillary action (Figure 1a). The driving force is the low humidity of the air, which leads to evaporation through exposed leaf surfaces.^36^ The one-cell-thick leaves are formed of a regular mesh of photosynthetic green cells and dead hyaline cells, among which one or both the upper and lower walls have pores of 4–8 μm in diameter to allow the free mass flow of water into and out from the cell.^36^ We expect that the small size of SMPs allows them to diffuse into the surface of mosses and transfer into the leaves (Figure 1a) through the capillaries (∼20 μm). Yet, it remains unknown whether bryophytes such as *Sphagnum* moss can take up (by uptake in this study, we mean passive diffusion of SMPs in moss) SMPs from the surrounding environment.

The objective of this study is twofold: (a) to quantify the adsorption of SMPs to the surfaces of peat in the presence and absence of NOM and (b) to understand how the chemical composition of SMPs and the presence of NOM on the particles influence their uptake by live *Sphagnum* moss and their impact on the composition and structure of microbiome. We use PVC-SMPs and PS-SMPs as model plastic particles. PS is a nonbiodegradable polymer that is produced for different applications, such as food packaging, electronics, and toys. PVC is a nonbiodegradable plastic that is used, e.g., in window frames, drainage pipes, and water service pipes. PS and PVC particles have been commonly identified in the environment,^37^ which are likely to be present also in peatlands. Quantification of SMPs in complex matrices such as peat and plants is challenging due to the limitations in the existing analytical methods to distinguish between synthesized and biogenic polymers.^38^ To facilitate tracking and quantification of SMPs in the culture media and organisms, a rare element, gadolinium (Gd), was entrapped in the structure of the particles (9.7–11%). Gd was used as a proxy for the quantification of SMPs via inductively coupled plasma mass spectrometry (ICP-MS), making their tracking possible even at the low concentrations^39^ likely to be present in nature.

## Materials and Methods

### Materials

The applied chemicals in this study were of reagent grade and purchased from Sigma-Aldrich. The SMP particles, including SP-SMPs (250 nm) and PVC-SMPs (250 nm) dispersed in Tween 20, were purchased from a commercial supplier CD-Bioparticles (NY 11967). Gd was entrapped in the particles upon our request. Milli-Q water was supplied by a Millipore filtration system (RiOs Essential 16 Water Purification System). The particles were originally stabilized with Tween 20.

### Particle Characterization

The hydrodynamic size of the particles and the zeta potential (ζ) were measured using a Zetasizer Nanodevice (Malvern Panalytical, Malvern, U.K.). The shape of the particles was determined using a transmission electron microscope (TEM), (JEOL JEM-2100F, JEOL Corp., Tokyo, Japan) operated at 200 kV. A scanning electron microscope (SEM), (Zeiss Sigma HD|VP, Carl Zeiss NTS, Cambridge, U.K.) was used with 4 kV for imaging the SMPs in plant tissues. Raman spectroscopy (Thermo Fisher Scientific, Madison, WI) was used to identify the polymer compositions of the SMPs (see Supporting Information Section 1). The hydrophobicity of the SMPs was measured after drying a droplet of the particle dispersion on aluminum surface and measuring the contact angle (A KSV Cam 200 contact angle) using Milli-Q (MQ) water at room temperature. The concentration of Gd ions and the number of particles in the exposure media (distilled water) and plant tissues were measured using an ICP-MS (PerkinElmer NexION 350D)^40^ operating on a single particle and standard mode. To test the presence of Gd in the particles, PS-SMPs and PVC-SMPs dispersions were deposited on silicon nitride windows (SiNx) and measured using X-ray fluorescence, which is a nondestructive analytical technique used to determine the elemental composition of materials (Supporting Information Section 2) at nm resolution.^41^

### Incubation of SMPs in NOM solution

The NOM used in this study was extracted from natural surface water in Finland (Lake Hietajärvi) as described in a previous study.^42^ The mixture of the SMPs with NOM was prepared as described previously.^26^ Briefly, 1 g of NOM solution was prepared in 100 mL of MQ water (Supporting Information Section 3) and used as stock solution. To provide the NOM-coated SMPs, 10 mg of the particles (PS-SMPs or PVC-SMPs) was mixed with 50 mg L^–1^ of NOM solution (pH 8) for 24 h at 20 °C to pre-condition them. The selected concentration of NOM represents the highest environmental level, reported by the FOREGS database for Finland Stream Water (reported as NPOC in the Stream Water data).^43^ The mixture was stirred for 24 h in the dark at room temperature using a magnet stirring. After 24 h, the dispersion was centrifuged (Multifuge 1S-R, Germany) at 3000*g* for 30 min at 4 °C to pellet the particles and to separate the NOM-SMPs from the NOM solution. The obtained dispersion was used for the experiments.

### Sorption Experiments

The adsorption experiments of SMPs to peat of moss were carried out in glass jars without live mosses so that 100 g (fresh weight) of dead *Sphagnum teres* shoots (peat) was put into every 200 mL glass jar and filled with distilled water. The dead mosses were collected from the same location where we sampled the alive mosses. Two more control samples were used only for the adsorption experiment, including SMPs and SMP-NOM in distilled water without peat to evaluate the sorption of the particles to the glass walls. The samples were placed under the same condition used for the moss exposure (see Supporting Information Section 4), which is described in the next section. Dispersions of approximately 10 mg L^–1^ SP-SMPs, PVC-SMPs, PS-SMP-NOM, and PVC-SMP-NOM were added to the system. The concentration of the particles was ∼10 mg L^–1^. The samples were gently mixed every day using a 10 mL pipette to minimize particle sedimentation. On days 1, 4, 8, 12, and 14, 1 mL of the water samples was taken from the top of the jars after mixing the water and then replaced with 1 mL of distilled water. We selected these time points because the exposure for the uptake experiment was performed for 14 days. The mass concentration of the Gd and the particle number in the water samples were measured using ICP-MS and spICP-MS, respectively (PerkinElmer NexION 350D) (see Supporting Information Section 5). The concentration of Gd and number of particles in the control samples without peat were also measured to ensure that the particles did not sediment or were not adsorbed to the glass walls. The reduced volume of water due to evaporation was replaced with distilled water 4 times per week.

### Developing the Mesocosms for Exposure Test

Mosses (*S. teres*) were collected from a mesotrophic spruce swamp forest in south-eastern Finland, middle-boreal zone (63°8′51.628″, 29°4′25.927′′). This species grows relatively high from the water table (10–15 cm) in habitats with a pH of 5–6. Mesocosms were constructed using 100 g (fresh weight) of dead *S. teres* shoots (peat) in 200 mL glass jars. The live *S. teres* (20 individuals) were added on top of the peat, and the jars were filled with distilled water to create water table above the peat. Note that the living mosses were washed with tap water and immediately added to the jars and cultured under controlled conditions, which represented the boreal summer conditions (see Supporting Information Section 4). Since the aim of this study was to compare the impact of SMP particles (PS-SMPs and PVC-SMPs) as a function of their polymer types (chemical composition) and presence/absence of NOM, we used an equal number of both particles in the exposure test. Since the concentration of SMPs in peatlands is unknown, we used particle numbers equal to the expected concentration of SMPs in the environment, which is reported to be ∼5 μg L^–1^.^44^ We used 5.1 and 5 μg L^–1^ of PS-SMPs and PVC-SMPs for the exposure test, respectively. The measured particle numbers in the exposure system were 2.0 × 10^9^, 1.5 × 10^9^, 2.5 × 10^9^, and 1.6 × 10^9^ per L of water for PS-SMPs, PS-SMP-NOM, PVC-SMPs, and PVC-SMP-NOM, respectively. After 1 month of culturing under controlled conditions, the water of the systems was replaced by the dispersions of the SMPs in distilled water. The treatments contain three replicates of each PS-SMPs, PVC-SMPs, PS-SMP-NOM, PVC-SMP-NOM, and control (without particles and NOM). The mesocosm-based exposure tests were performed for 14 days. To minimize the sedimentation of the particles in the jars, the water of each mesocosm was gently mixed using a 10 mL pipette every 24 h. The reduced volume of water due to evaporation was replaced with distilled water four times per week.

### Quantification of SMPs in Mosses’ Tissues

To quantify the SMPs in the plant tissue, a method was developed and validated (in-house) to extract the particles from the plants (see Supporting Information Section 5). Then, we quantified the concentration of Gd ions and the number of particles in the samples using spICP-MS (Table S1)

### SMPs Observation Using a Scanning Electron Microscope

All of the observations using an electron microscope have been done on the live moss after exposure. To observe the SMPs in mosses, the samples were cut into 3–5 μm slices using a microtome and fixed using 2.5% glutaraldehyde in 0.1 M phosphate buffer pH 7.4. The sections were dehydrated and coated with a thin layer of gold (30 nm) using an Agar Auto Sputter Coater, to ensure electrical conductivity on the sample surfaces and to minimize or eliminate surface charging. A field emission (Schottky type) SEM was used to observe the samples and find the particles. During the observation, an acceleration voltage of 4 kV was used under high vacuum conditions (pressure, *P* < 2 mPa). The micrographs were captured with an InLens secondary electron detector to maximize the spatial resolution and to visualize all of the particles of interest.

### Bacterial Identification

The dead moss (peat), which was used as bases for alive mosses to grow, was collected and washed. Around 500 mg of peat was separated using sterile scissors into a 50 mg conical vial containing 25 mL of epiphyte removal buffer (see Supporting Information Section 6). The samples were centrifuged (10 min at 4 °C, 4000*g*), and the supernatants were discarded and stored at −80 °C for DNA extraction. The extracted DNA was used for high-throughput DNA sequencing. The distribution and relative abundances of epiphytic bacteria from root samples were assessed by sequencing triplicate samples for each treatment. The V3–V4 hypervariable region of the 16S rRNA genes was amplified, using primer pair 341F (CCTAYGGGRBGCASCAG) and 806R (GGACTACNNGGGTATCTAAT). Sequencing was done by Novogene Company Limited (Cambridge, United Kingdom). The 16S rRNA gene amplicons were selected by agarose gel electrophoresis (2%), purified, pooled, and sequenced on paired-end illumina Novaseq. 6000 platform. Sequences were assigned to samples based on their unique barcodes. The primer sequences and barcodes were trimmed from the paired-end reads to remove low-quality regions. Paired-end reads were merged using FLASH (V1.2.7). Quality filtering on sequence reads was performed in Qiime, (V1.7.0) to obtain high-quality clean sequences. UCHIME software was used for the detection and removal of chimera sequences. The 16S rRNA gene sequence data were submitted to the National Centre for Biotechnology Information (NCBI) under BioProject ID PRJNA789126.

### Data Analysis

SigmaPlot 14 was used to analyze the data. SigmaPlot 14, Microsoft office 2022, and OriginLab (OriginPro 8.5) were used to plot the graphs. Data were evaluated statistically for normality using a Shapiro–Wilk test. *T-test* was used to determine statistically significant differences between the number of PS-SMPs and PVC-SMPs in shoot and leaves. The data was reported as mean ± standard deviation. Bacterial sequence analysis was performed by Uparse software,^45^ Uparse v7.0.1090. Sequences with ≥97% similarity were assigned to the same operational taxonomic units (OTUs). For each representative sequence, Qiime in Mothur method was performed against the database of SILVA138 Database^46^ for species annotation. The phylogenetic relationship of all OTUs representative sequences was obtained by MUSCLE (Version 3.8.31). The biodiversity of each sample was analyzed using OTUs, and Goods coverage. By principal coordinate analysis (PCoA), dominance, Simpson, Shannon, and evenness indices were calculated using Past 3 software (Version 3.20).^47^ Common and shared OTUs of epiphytic bacterial communities between different treatments were identified using the Venn Diagram package in R.

## Results and Discussion

### Characterization of SMP Particles

In this study, we used spherical PS- and PVC-SMPs of 250 nm (polydispersity index: 0.2) particle size stabilized with Tween 20. The PS-SMPs (contact angle of 80 ± 2) were more hydrophobic than the PVC-SMPs (contact angle of 60 ± 1) as determined using their contact angles with water. The hydrodynamic diameter of the synthesized PS-SMPs and PVC-SMPs particles was 256 ± 6 and 243 ± 8 nm as determined by dynamic light scattering (DLS), respectively. The measured ζ of the PS-SMPs and PVC-SMPs in water was −15 ± 1 and −15 ± 3 mV, respectively.

The hydrodynamic size for NOM-coated SMPs was 453 ± 24 and 416 ± 48 nm for PS-SMP-NOM and PVC-SMP-NOM, respectively, which shows a significant increase in size (*t*-*test, p* < 0.05) due to the sorption of NOM on the particle surface, compared to the naked particles. The ζ value of the PS-SMP-NOM and PVC-SMP-NOM decreased to −17 ± 3 and −19 ± 2 mV, respectively. These negative ζ values can increase the repulsion between the particles and consequently increase the particles’ stability in the systems.

### Stability of the SMPs

First, we tested the presence of Gd in the particles. The elemental map of the SMP particles revealed the presence of Gd (blue color) in the agglomerates of the particles (Figure 1b).

The cell walls of moss exhibit a high cation exchange capacity, where cations such as NH_4_^+^, Ca^2+^, Mg^2+^, and K^+^ are absorbed from the surrounding water in exchange for H^+^. This mechanism decreases the pH of the surrounding water to a level as low as 3.3.^35^ We investigated the stability of the particles in pH 3.5–4 for 14 days by measuring the number of the SMPs that contain Gd and the quantity of the released Gd ions from the particles using single-particle (sp)ICP-MS.^48^ This technique allows the differentiation between the ionic and particulate forms of metals.^40^ No Gd ions could be detected after 14 days of incubation, and no significant difference (*t*-*test, p* > 0.05) was found between the number of particles on day 1 and the number of particles on day 14 (Figure 1c). This confirms the stability of the particles against degradation at pH 4.

Agglomeration of particles over time might influence the uptake and toxicity of the SMPs. To ensure that the particles were stable against agglomeration, the SMPs were dispersed in distilled water. The hydrodynamic size of the particles was measured over 14 days using DLS. The results showed that both particles were stable against agglomeration in water (Figure 1d).

### Sorption of SMPs to the Peat Surfaces

Before investigating the adverse effects of SMPs on the plant and bacteria of the peatland, it is critical to understand the behavior of the particles in peat structures. This allows us to understand the stability and bioavailability of the particles in the ecosystem. We tested the sorption of the particles to the peat surface. Note that the setup contained peat without live *Sphagnum* moss (to avoid uptake by live plants) and dispersions of 10 mg L^–1^ of PS-SMPs, PS-SMP-NOM, PVC-SMPs, or PVC-SMP-NOM in distilled water. This concentration of SMPs was used only for the sorption experiment and not for the exposure test because it allows measuring small changes (i.e., μg L^–1^) in the concentration of Gd (as proxy of the SMPs) and particle characterization over time. The measured concentrations by spICP-MS (before the sorption experiment) for PS-SMP, PS-SMP-NOM, PVC-SMPs, and PVC-SMP-NOM were 5.3 × 10^11^, 8.6 × 10^11^, 5 × 10^11^, and 3 × 10^11^, respectively.

**Figure 2:**
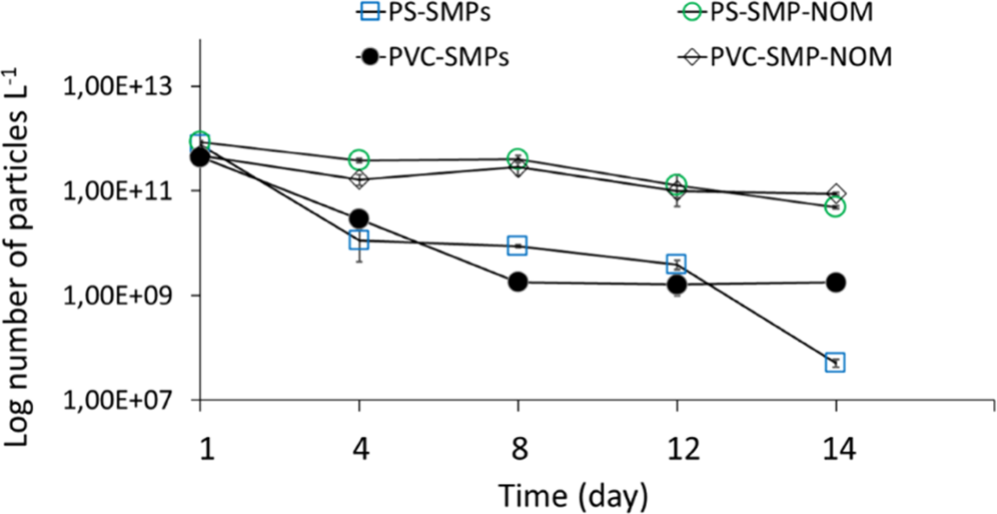
Sorption of SMPs to the peat surface over time by measuring the number of particles in the water on days 1, 4, 8, 12, and 14 using spICP-MS.

The spICP-MS data revealed a decrease in the number of the PS-SMPs and PVC-SMPs over 14 days of mixing (Figure 2). The total mass concentration of Gd over time showed the same trend as measured for the particle number (Figure S1, Supporting Information). These findings suggest that the particles were adsorbed to the peat surface. To ensure that the particles were not attached to the glass walls of the mesocosms, we performed a control experiment without the peat. No significant decreases were observed for the number of particles over time in the absence of the peat in the control samples (Figure S2, Supporting Information). No Gd ion could be detected in the test samples for both particles, indicating that no Gd ions were released from the particles in the presence of peat.

The presence of NOM on the surface of the particles decreased the sorption of both particles to peat regardless of the chemical composition of the SMPs (Figure 2). This confirms our hypothesis that the steric repulsion between the peat surfaces and NOM-coated SMP decreases the sorption of the particles to the peat. This is of paramount importance for environmental risk assessment of plastic because higher sorption of SMPs (without NOM) to peat might increase the particle retention time in the peat and turn peatlands to sink for plastic particles. The retention of SMPs might influence the uptake of the particles by the alive moss and the composition of the bacteria (these hypotheses have been tested in the next sections). It is, however, unlikely that the SMPs remain pristine without NOM in peatland ecosystems, which permanently contain a considerable amount of dissolved NOM.^49^ From an environmental safety perspective, the presence of NOM can increase the dispersion of SMPs and may increase the bioavailability of these particles to (micro)organisms as reported for engineered nanomaterials.^50,51^

### Quantifying the Uptake of SMPs by Live *Sphagnum* Moss

**Figure 3:**
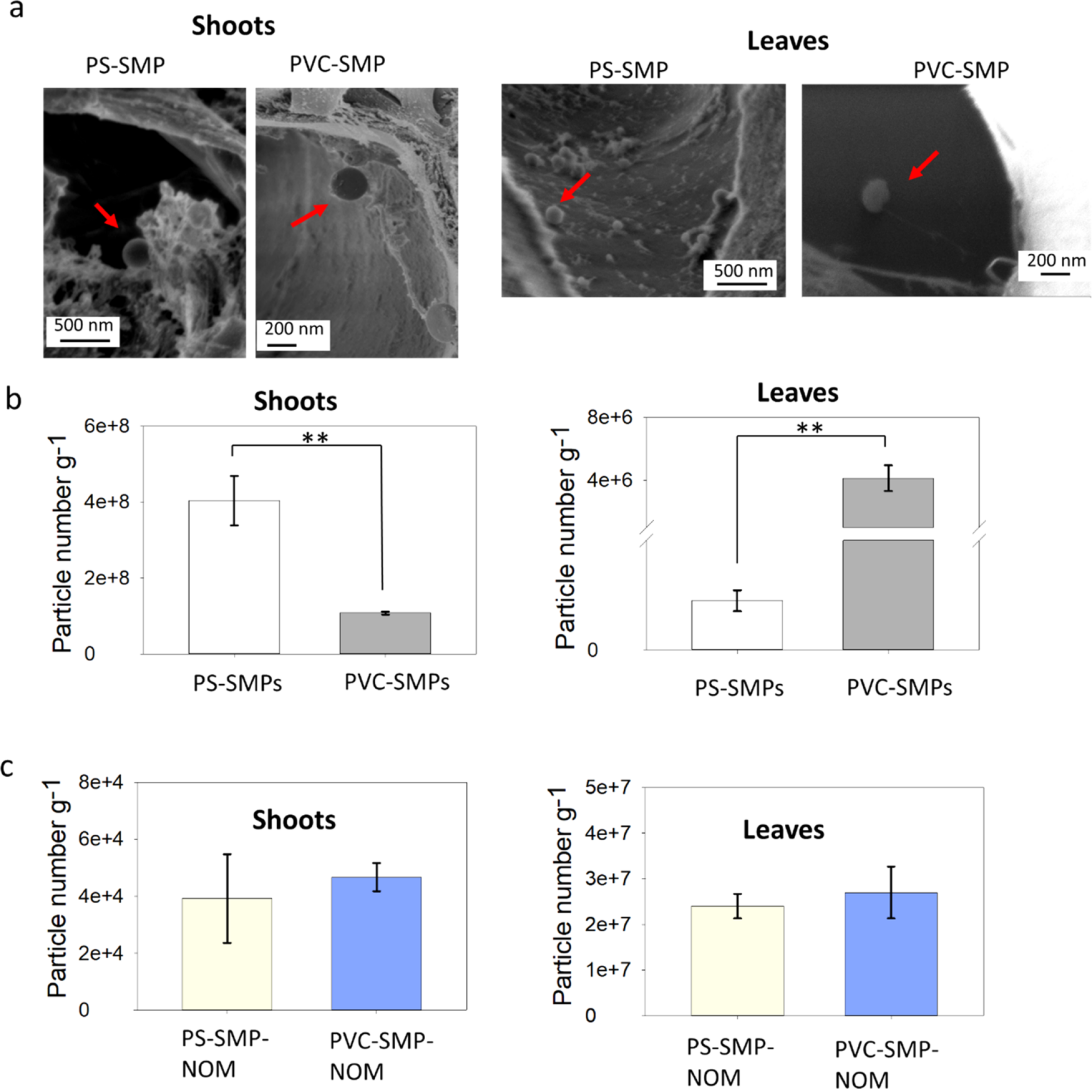
(a) SEM images of PS-SMPs and PVC-SMPs in the shoot and leaves of the moss after 14 days of exposure. The red arrows indicate the location of the particles. (b) Number of SMPs in the shoot and leaves of moss, as measured by spICP-MS after 14 days of exposure. (c) Number of PS-SMP-NOM and PVC-SMP-NOM in the shoot and leaves of *Sphagnum* moss as measured by spICP-MS.

We further investigated the uptake of SMPs (naked and NOM-coated SMPs) by the plants (*Sphagnum* moss). Note that, in this study, we did not investigate the influence of particle size on the uptake. We have focused only on particle chemistry by keeping all other parameters like size equal between the two particles (i.e., PS-SMPs and PVC-SMPs). Water movement in moss is facilitated by capillary action, which may allow the movement of the particles through the peat and subsequent uptake of the particles by the plants. The obtained SEM images confirmed that both PS-SMPs and PVC-SMPs penetrate the *S. teres* and occur in the shoots and leaves (Figure 3a). We determined the quantities of the particles in each plant tissue, to understand how the polymer type influences the accumulation of the particles in the tissues. Accordingly, the SMPs were first extracted from the tissues and then quantified using spICP-MS to measure the number of particles in each plant tissue. No particles could be detected in the control samples (controls with moss but no particles and NOM and controls with moss and NOM but no particles) and no Gd ions could be detected in the moss tissues.

Despite being exposed to an equal number of particles, a significantly (*t*-*test*, *p* < 0.01) higher number of PS-SMPs accumulated in the moss’ shoots compared to PVC-SMPs (Figure 3b). On the contrary, the number of PS-SMPs in the leaves was significantly (*t*-*test*, *p* < 0.01) lower than the number of PVC-SMPs (Figure 3b). One explanation is that the higher hydrophobicity^20^ of PS-SMPs (contact angle of 80 ± 2) compared to PVC-SMPs (contact angle of 60 ± 1) increases their interaction with the plant cells, which in turn could lead to a slower movement and higher retention of PS-SMPs in the moss shoot while transferring by the capillary force. A previous study showed that PS-SMPs accumulate in lettuce shoots.^39^ Our findings confirm that the type of polymer influences the uptake and distribution of plastic particles in *Sphagnum* moss. Although the mass of accumulated PS-SMP has been reported previously in plants,^32,39^ this study is the first to report the uptake of plastic particles in plants on a particle number basis.

The SEM images also confirmed that the NOM-coated SMPs were taken up by the moss’ shoots (Supporting Information Figure S3), but we did not observe NOM-coated SMPs inside the leaves. We, however, observed NOM-coated PVC-SMPs on the external surface of the leaves close to the pores (Supporting Information Figure S4). It is likely that the PVC-SMPs were transferred by water using the capillary force and excreted from the pores, where they aggregate after evaporation of water from the surface of the leaves. This need to be investigated in more detail in future studies because this indicates that moss could facilitate the transfer of SMP particles from water and soil into the atmosphere after excretion from the pores on their leaves.

When the particles are coated with NOM, the uptake by moss decreased compared to the naked particles (*t*-*test*, *p* < 0.05) regardless of the chemical composition (type) of the particles in both tissues (Figure 3c). Previous studies on metallic nanomaterials have shown that the presence of NOM on the surface of particles could decrease the uptake of SMP particles by algae.^51^ Attachment of NOM to the surface of the particles can lead to electrostatic and steric repulsion between the particles and the plant cell wall, reducing the contact between SMPs and the plant, as reported for metallic nanoparticles.^52^ This indicates that despite increasing the dispersion stability of SMPs, NOM decreased the uptake of the PS- and PVC-SMPs by moss. This finding might be applicable to other plant species and algae, which need to be tested in future studies.

Interestingly, there were no significant differences (*t*-*test*, *p* > 0.05) between the uptake of NOM-coated PS-SMPs and NOM-coated PVC-SMPs in both shoots and leaves. This finding shows that NOM coating on the surface of plastic particles plays an important role in the fate of the particles by masking the surface properties of SMPs, thus influencing their uptake and biodistribution.

### Effects of SMPs on the Microbiome

**Figure 4:**
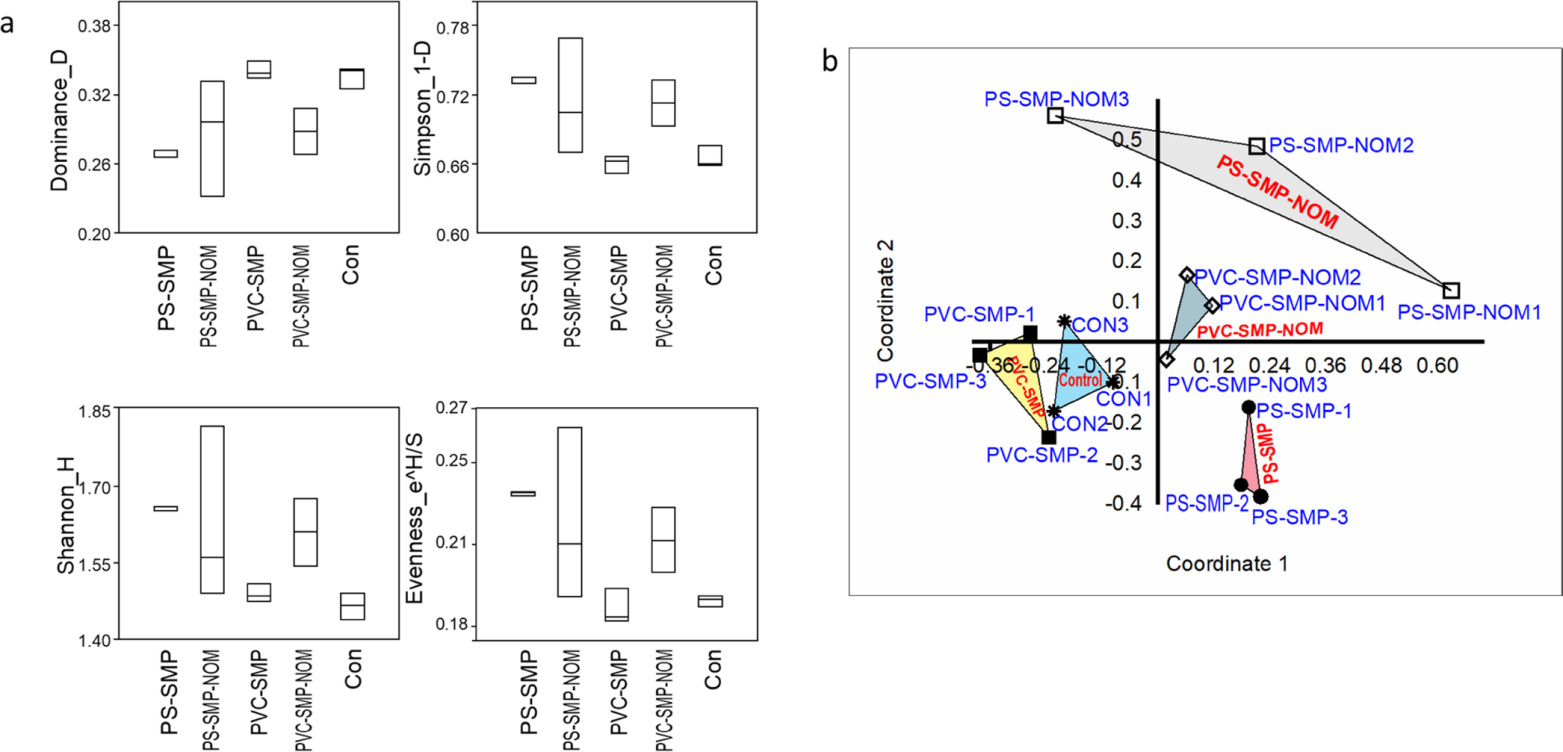
(a) Dominance index, Simpson index, Shannon diversity index, and evenness index showing the bacterial diversity in different treatments. (b) Principal coordinates analysis (PCoA) of the bacterial communities based on OTU levels representing the similarity matrix generated by cluster analysis.

Next, we investigated the influence of the environmentally relevant concentration of SMPs on the peatland bacterial communities in the mesocosms. To estimate the α diversity of the bacterial communities, we calculated the number of OTUs and dominance, evenness, Shannon, and Simpson indices. Sequence analyses showed high estimated coverage of 99% (goods coverage of 0.99) from all samples indicating near-complete sequencing of the entire bacterial community members. The saturation of rarefaction curves indicated that bacterial communities were sufficiently deep sequenced (Figure S5, Supporting Information). The alpha diversity analysis highlighted the rich taxonomic diversity in the control (without particles) and treated samples. The dominance index of samples ranged from 0.23 to 0.35, showing the dominance of fewer bacterial groups at higher values. The Simpson index of samples ranged from 0.66 to 0.76. This shows the presence of diverse microbial communities in all samples. The Shannon index varied from 1.45 to 1.80, and the evenness index ranged from 0.184 to 0.264, emphasizing the richness of bacterial species in the control and SMP-treated samples (Figure 4a).

Bacterial diversity index and *Tukey’s post hoc test* within naked particles, i.e., PS-SMPs and PVC-SMPs and NOM-coated particles, i.e., PS-SOM and PVC-NOM, did not show any significant (*Tukey’s post hoc test*, *p* < 0.05) variations. Although, when we compared all of the treatments, higher Shannon values and significant differences (*Tukey’s post hoc test*, *p* < 0.05) were detected in NOM-coated SMPs (PS-SMP-NOM and PVC-SMP-NOM) than in the naked particles. This indicates that bacterial diversity was increased significantly in the presence of NOM on the surface of SMPs. This is the first study showing that the presence of NOM on the surface of plastic particles could influence bacterial diversity. PcoA confirms that the bacterial community structure is significantly influenced when exposed to NOM-coated SMPs compared to naked particles (Figure 4b). There is no information on the effects of plastic particles on the microbiome in peatland ecosystems; thus, we compared our findings with results from other ecosystems. Our findings agree with earlier results^53,54^ documenting that the richness of microbial communities in freshwater varies when exposed to PS-SMPs.

We used a Venn diagram to examine the presence of core bacterial communities, core defined as the shared microbial communities and represented by overlapping circles at 97% identity (Supporting Information Figure S6). We identified 1762, 1576, 1766, 1817, and 1796 OTUs in control (without particles), PS-SMPs, PS-SMP-NOM, PVC-SMP, and PVC-SMP-NOM, respectively. As shown in Figure S6, 1000 OTUs were shared among all samples. The unique OTU numbers for control, PS-SMPs, PS-SMP-NOM, PVC-SMP, and PVC-SMP-NOM were 87, 83, 155, 184, and 130, respectively. Among all of the treatments, PVC-SMPs had the highest unique OTUs. When compared with the treatment within naked particles (PS-SMPs and PVC-SMPs) and NOM-coated particles (PS-SMP-NOM and PVC-SMP-NOM), the highest number of unique OTUs were found in PVC-SMPs and PS-SMP-NOM, respectively. These findings suggest that both the chemistry of the particles and the presence of NOM can influence the bacterial community.

Sequencing analysis detected a total of 23 phyla with 56 classes, 160 orders, and 251 families. Taxonomic affiliation of bacterial communities at the phylum level revealed a high abundance of seven major phyla in all samples: *Proteobacteria* (46.59–54%), *Acidobacteriota* (11.63–19.67%), *Firmicutes* (5.09–12.59%), *Actinobacteriota* (5.97–9.89%), *Verrucomicorbiota* (2.83–6.07%), *Bacteroidota* (2.85–4.45%), and *Cyanobacteria* (0.63–3.01%). Our results are in line with previous studies^55,56^ which report that bacterial communities of peatlands are mainly composed of the phyla *Proteobacteria*, *Acidobacteria*, *Bacteroidetes*, *Actinobacteria*, and *Firmicutes*. Other phyla such as *Patescibacteria*, *Desulfobacterota*, *Spirochaetota*, *Elusimicrobiota*, *Planctomycetota*, and *Armatimonadota* were present in lower numbers and contributed to only 4% of the total sequence numbers obtained from this study. The phylum-level relative abundance was further investigated at the family level (Figures S7, Supporting Information). The top 5 families identified across the whole dataset included *Acetobacteraceae* (16.08–21.11%), *WD260* (7.54–10.87%), *Acidobacteriaceae* (6.38–12.03%), *Bifidobacteriaceae* (1.56–6.63%), and Subgroup_2 (2.29–5.46%) with a significant number of sequences affiliated to unidentified taxa (6.84–9.94%). At a higher phylogenetic resolution, the diversity of dominating bacteria increased, but the overall pattern remained the same (Figures S7, Supporting Information).

Exposure to PS-SMPs (naked and NOM-coated particles) decreased the relative abundance of *Cyanobacteria*, *Acidobacteriota*, and *Verrucomicorbiota* and increased the relative abundance of *Proteobacteria*, *Actinobacteriota*, *Firmicutes*, and *Bacteroidota*. After exposure to PVC-SMPs (naked particles), the relative abundance of *Proteobacteria*, *Acidobacteriota*, and *Bacteroidota* decreased and the relative abundance of *Cyanobacteria*, *Firmicutes*, *Actinobacteriota*, and *Verrucomicorbiota* increased compared to the control. After exposure to NOM-coated PVC-SMPs, the relative abundance of *Cyanobacteria* diminished, whereas abundances of *Proteobacteria*, *Firmicutes*, *Actinobacteriota*, *Verrucomicorbiota*, and *Bacteroidota* were enhanced in comparison to the control. Our findings show that there is a certain degree of variation in the abundance and diversity of bacterial communities when exposed to SMPs in comparison to the control. Although some previous studies have reported varying influence of PVC microplastics on soil microbial community composition^57,58^ and oligochaete gut samples,^59^ to our knowledge, none of the earlier studies have investigated the effect of SMPs of different chemical compositions and their NOM coating on bacterial communities in peatlands. Yet, it has been demonstrated^54^ that the microbial community richness of freshwater decreased when exposed to nano-sized PS particles.

We conclude that the physicochemical properties of peat and SMP facilitate the adsorption of plastic particles. This suggests that peatland ecosystems could adsorb plastic particles. The load of plastic particles in peatland ecosystems is unknown yet. The limitation in analytical techniques might currently hinder monitoring of the true extent of plastic pollution in peatland ecosystems. Thus, we recommend future studies focus more on the possible adverse effects of plastic pollution in these ecosystems. Although in this study moss was exposed to similar numbers of PS-SMPs and PVC-SMPs, the uptake of the particles by the plants and their biodistribution in the plants’ tissues were different. This suggests that the generated data on one type of plastic might not be transferable to other types of plastics because the chemical composition of plastics influences how they behave in peatland and interact with (micro)organisms in these ecosystems. Other physicochemical properties such as size and shape also may play important roles in the bioavailability and biodistribution of SMPs in not only peatland organisms but also organisms from other ecosystems. This might complicate the risk assessment of plastic particles, as reported for nanomaterials. Our findings demonstrate that the transformation of SMPs due to the sorption of NOM can dramatically change their sorption behavior, uptake, and biodistribution. Moreover, we recommend future studies consider the aging (e.g., transformation) process of SMPs for the assessment of different aspects of environmental risks. Finally, our findings show that the naked SMPs and the NOM-covered SMP influence the relative abundance and diversity of the bacterial communities of the peatland ecosystem, which could possibly influence ecosystem functioning.

## Supporting Information Available

Raman spectroscopy (Section 1); X-ray fluorescence (XRF) microscopy (Section 2); NOM preparation (Section 3); sorption of SMPs to the peat surfaces (Section 4); ICP-MS and spICP-MS measurement (Section 5); Bacterial identification (Section 6); uptake of NOM-coated SMPs by the shoots (Section 7); presence of SMPs on the external surface of the leaves (Section 8); sorption of SMPs to the peat surface over time (Figure S1); number of particles on days 1 and 14 in the jars without peats (Figure S2); SEM images of PS-SMP-NOM and PVC-SMP-NOM (Figure S3); SEM image of PVC-SMPs (Figure S4); saturation of rarefaction curves (Figure S5); Venn diagram indicating common and unique OTUs (Figure S6); percentages of relative abundances of major and minor taxa of bacterial communities (Figure S7); single-particle inductively coupled plasma mass spectrometry setting (Table S1); ICP-MS setup (Table S2); ICP-MS operating conditions (Table S4); (Figure S1) (PDF)

## References

[ref1] Xu J.; Morris P. J.; Liu J.; Holden J. PEATMAP: Refining Estimates of Global Peatland Distribution Based on a Meta-Analysis. CATENA 2018, 160, 134–140. 10.1016/j.catena.2017.09.010.

[ref2] Raghoebarsing A. A.; Smolders A. J. P.; Schmid M. C.; Irene W.; Rijpstra C.; Wolters-Arts M.; Derksen J.; Jetten M. S. M.; Schouten S.; Sinninghe Damsté J. S.; Lamers L. P. M.; Roelofs J. G. M.; Op Den Camp H. J. M.; Strous M. Methanotrophic Symbionts Provide Carbon for Photosynthesis in Peat Bogs. Nature 2005, 436, 1153–1156. 10.1038/nature03802. 16121180

[ref3] LAI D. Y. F. Methane Dynamics in Northern Peatlands: A Review. Pedosphere 2009, 19, 409–421. 10.1016/S1002-0160(09)00003-4.

[ref4] Batjes N. H. Total Carbon and Nitrogen in the Soils of the World. Eur. J. Soil Sci. 2014, 65, 10–21. 10.1111/ejss.12114_2.

[ref5] Turetsky M. Current Disturbance and the Diminishing Peatland Carbon Sink. Geophys. Res. Lett. 2002, 29, 1526 10.1029/2001GL014000.

[ref6] Swindles G. T.; Morris P. J.; Mullan D. J.; Payne R. J.; Roland T. P.; Amesbury M. J.; Lamentowicz M.; Turner T. E.; Gallego-Sala A.; Sim T.; Barr I. D.; Blaauw M.; Blundell A.; Chambers F. M.; Charman D. J.; Feurdean A.; Galloway J. M.; Gałka M.; Green S. M.; Kajukało K.; Karofeld E.; Korhola A.; Lamentowicz Ł.; Langdon P.; Marcisz K.; Mauquoy D.; Mazei Y. A.; McKeown M. M.; Mitchell E. A. D.; Novenko E.; Plunkett G.; Roe H. M.; Schoning K.; Sillasoo Ü.; Tsyganov A. N.; van der Linden M.; Väliranta M.; Warner B. Widespread Drying of European Peatlands in Recent Centuries. Nat. Geosci. 2019, 12, 922–928. 10.1038/s41561-019-0462-z.

[ref7] HELCOM. European Red List of Habitats

[ref8] Couillard D. The Use of Peat in Wastewater Treatment. Water Res. 1994, 28, 1261–1274. 10.1016/0043-1354(94)90291-7.

[ref9] Ovenden L. Peat Accumulation in Northern Wetlands. Quat. Res. 1990, 33, 377–386. 10.1016/0033-5894(90)90063-Q.

[ref10] Mckay G. Peat for Environmental Applications: A Review. Dev. Chem. Eng. Miner. Process. 2008, 4, 127–155. 10.1002/apj.5500040302.

[ref11] Cojocaru C.; Macoveanu M.; Cretescu I. Peat-Based Sorbents for the Removal of Oil Spills from Water Surface: Application of Artificial Neural Network Modeling. Colloids Surf., A 2011, 384, 675–684. 10.1016/J.COLSURFA.2011.05.036.

[ref12] Glooschenko W. A.; Capobianco J. A. Trace Element Content of Northern Ontario Peat. Environ. Sci. Technol. 1982, 16, 187–188. 10.1021/es00097a012.

[ref13] Materić D.; Ludewig E.; Brunner D.; Röckmann T.; Holzinger R. Nanoplastics Transport to the Remote, High-Altitude Alps. Environ. Pollut. 2021, 288, 117697 10.1016/j.envpol.2021.117697. 34273766

[ref14] Redondo-Hasselerharm P. E.; Gort G.; Peeters E. T. H. M.; Koelmans A. A. Nano- and Microplastics Affect the Composition of Freshwater Benthic Communities in the Long Term. Sci. Adv. 2020, 6, eaay4054 10.1126/sciadv.aay4054. 32064347 PMC6994214

[ref15] Horton A. A.; Barnes D. K. A. Microplastic Pollution in a Rapidly Changing World: Implications for Remote and Vulnerable Marine Ecosystems. Sci. Total Environ. 2020, 738, 140349 10.1016/j.scitotenv.2020.140349. 32806379

[ref16] Ajith N.; Arumugam S.; Parthasarathy S.; Manupoori S.; Janakiraman S. Global Distribution of Microplastics and Its Impact on Marine Environment–a Review. Environ. Sci. Pollut. Res. 2020, 27, 25970 10.1007/s11356-020-09015-5. 32382901

[ref17] Rillig M. C. Microplastic in Terrestrial Ecosystems and the Soil?. Environ. Sci. Technol. 2012, 46, 6453–6454. 10.1021/es302011r. 22676039

[ref18] Brahney J.; Hallerud M.; Heim E.; Hahnenberger M.; Sukumaran S. Plastic Rain in Protected Areas of the United States. Science 2020, 368, 1257–1260. 10.1126/science.aaz5819. 32527833

[ref19] Rillig M. C.; Lehmann A. Microplastic in Terrestrial Ecosystems. Science 2020, 368, 1430–1431. 10.1126/science.abb5979. 32587009 PMC7115994

[ref20] Min K.; Cuiffi J. D.; Mathers R. T. Ranking Environmental Degradation Trends of Plastic Marine Debris Based on Physical Properties and Molecular Structure. Nat. Commun. 2020, 11, 727 10.1038/s41467-020-14538-z. 32024839 PMC7002677

[ref21] Bancone C. E. P.; Turner S. D.; Ivar do Sul J. A.; Rose N. L. The Paleoecology of Microplastic Contamination. Front. Environ. Sci. 2020, 8, 1–20. 10.3389/fenvs.2020.574008.

[ref22] Allen D.; Allen S.; Le Roux G.; Simonneau A.; Galop D.; Phoenix V. R. Temporal Archive of Atmospheric Microplastic Deposition Presented in Ombrotrophic Peat. Environ. Sci. Technol. Lett. 2021, 8, 954–960. 10.1021/acs.estlett.1c00697. 34778488 PMC8582260

[ref23] Pradel A.; Ferreres S.; Veclin C.; El Hadri H.; Gautier M.; Grassl B.; Gigault J. Stabilization of Fragmental Polystyrene Nanoplastic by Natural Organic Matter: Insight into Mechanisms. ACS ES&T Water 2021, 1, 1198–1208. 10.1021/acsestwater.0c00283.

[ref24] Mensch A. C.; Hernandez R. T.; Kuether J. E.; Torelli M. D.; Feng Z. V.; Hamers R. J.; Pedersen J. A. Natural Organic Matter Concentration Impacts the Interaction of Functionalized Diamond Nanoparticles with Model and Actual Bacterial Membranes. Environ. Sci. Technol. 2017, 51, 11075–11084. 10.1021/acs.est.7b02823. 28817268

[ref25] Arenas-Lago D.; Abdolahpur Monikh F.; Vijver M. G.; Peijnenburg W. J. G. M. Dissolution and Aggregation Kinetics of Zero Valent Copper Nanoparticles in (Simulated) Natural Surface Waters: Simultaneous Effects of PH, NOM and Ionic Strength. Chemosphere 2019, 226, 841–850. 10.1016/j.chemosphere.2019.03.190. 30974377

[ref26] Abdolahpur Monikh F.; Vijver M. G.; Guo Z.; Zhang P.; Darbha G. K.; Peijnenburg W. J. G. M. Metal Sorption onto Nanoscale Plastic Debris and Trojan Horse Effects in Daphnia Magna: Role of Dissolved Organic Matter. Water Res. 2020, 186, 116410 10.1016/j.watres.2020.116410. 32932097

[ref27] Andersen R.; Chapman S. J.; Artz R. R. E. Microbial Communities in Natural and Disturbed Peatlands: A Review. Soil Biol. Biochem. 2013, 57, 979–994. 10.1016/j.soilbio.2012.10.003.

[ref28] Elliott D. R.; Caporn S. J. M.; Nwaishi F.; Nilsson R. H.; Sen R. Bacterial and Fungal Communities in a Degraded Ombrotrophic Peatland Undergoing Natural and Managed Re-Vegetation. PLoS One 2015, 10, e0124726 10.1371/journal.pone.0124726. 25969988 PMC4430338

[ref29] Rydin H.; Barber K. E. Long-Term and Fine-Scale Coexistence of Closely Related Species. Folia Geobotanica 2001, 36, 53–61. 10.1007/BF02803138.

[ref30] Breeuwer A.; Robroek B. J. M.; Limpens J.; Heijmans M. M. P. D.; Schouten M. G. C.; Berendse F. Decreased Summer Water Table Depth Affects Peatland Vegetation. Basic Appl. Ecol. 2009, 10, 330–339. 10.1016/j.baae.2008.05.005.

[ref31] Field C. D.; Dise N. B.; Payne R. J.; Britton A. J.; Emmett B. A.; Helliwell R. C.; Hughes S.; Jones L.; Lees S.; Leake J. R.; Leith I. D.; Phoenix G. K.; Power S. A.; Sheppard L. J.; Southon G. E.; Stevens C. J.; Caporn S. J. M. The Role of Nitrogen Deposition in Widespread Plant Community Change Across Semi-Natural Habitats. Ecosystems 2014, 17, 864–877. 10.1007/s10021-014-9765-5.

[ref32] Sun X.-D.; Yuan X.-Z.; Jia Y.; Feng L.-J.; Zhu F.-P.; Dong S.-S.; Liu J.; Kong X.; Tian H.; Duan J.-L.; Ding Z.; Wang S.-G.; Xing B. Differentially Charged Nanoplastics Demonstrate Distinct Accumulation in Arabidopsis Thaliana. Nat. Nanotechnol. 2020, 15, 755–760. 10.1038/s41565-020-0707-4. 32572228

[ref33] Proctor M. C. F.; Tuba Z. Poikilohydry and Homoihydry: Antithesis or Spectrum of Possibilities?. New Phytologist. 2002, 156, 327–349. 10.1046/j.1469-8137.2002.00526.x. 33873572

[ref34] Moore P. D. The Ecology of Peat-Forming Processes: A Review. Int. J. Coal Geol. 1989, 12, 89–103. 10.1016/0166-5162(89)90048-7.

[ref35] Rice S. K. Mosses (Bryophytes). In Encyclopedia of Inland Waters, Elsevier Inc, 2009; pp 88–96.

[ref36] Hayward P. M.; Clymo R. S. Profiles of Water Content and Pore Size in Sphagnum and Peat, and Their Relation to Peat Bog Ecology. Proc. R. Soc. London, Ser. B 1982, 215, 299–325. 10.1098/rspb.1982.0044.

[ref37] Brandon J. A.; Jones W.; Ohman M. D. Multidecadal Increase in Plastic Particles in Coastal Ocean Sediments. Sci. Adv. 2019, 5, eaax0587 10.1126/sciadv.aax0587. 31517049 PMC6726453

[ref38] Abdolahpur Monikh F.; Vijver M. G.; Mitrano D. M.; Leslie H. A.; Guo Z.; Zhang P.; Lynch I.; Valsami-Jones E.; Peijnenburg W. J. G. M. The Analytical Quest for Sub-Micron Plastics in Biological Matrices. Nano Today 2021, 41, 101296 10.1016/j.nantod.2021.101296.

[ref39] Luo Y.; Li L.; Feng Y.; Li R.; Yang J.; Peijnenburg W. J. G. M.; Tu C. Quantitative Tracing of Uptake and Transport of Submicrometre Plastics in Crop Plants Using Lanthanide Chelates as a Dual-Functional Tracer. Nat. Nanotechnol. 2022, 17, 424 10.1038/s41565-021-01063-3. 35058654

[ref40] Abdolahpur Monikh F.; Chupani L.; Arenas-Lago D.; Guo Z.; Zhang P.; Darbha G. K.; Valsami-Jones E.; Lynch I.; Vijver M. G.; van Bodegom P. M.; Peijnenburg W. J. G. M. Particle Number-Based Trophic Transfer of Gold Nanomaterials in an Aquatic Food Chain. Nat. Commun. 2021, 12, 899 10.1038/s41467-021-21164-w. 33563998 PMC7873305

[ref41] Guo Z.; Zhang P.; Chakraborty S.; Chetwynd A. J.; Abdolahpur Monikh F.; Stark C.; Ali-Boucetta H.; Wilson S.; Lynch I.; Valsami-Jones E. Biotransformation Modulates the Penetration of Metallic Nanomaterials across an Artificial Blood-Brain Barrier Model. Proc. Natl. Acad. Sci. U.S.A. 2021, 118, 1–10. 10.1073/pnas.2105245118. PMC8285959 34260400

[ref42] Vogt R. D.; Akkanen J.; Andersen D. O.; Brüggemann R.; Chatterjee B.; Gjessing E.; Kukkonen J. V. K.; Larsen H. E.; Luster J.; Paul A.; Pflugmacher S.; Starr M.; Steinberg C. E. W.; Schmitt-Kopplin P.; Zsolnay Á. Key Site Variables Governing the Functional Characteristics of Dissolved Natural Organic Matter (DNOM) in Nordic Forested Catchments. Aquat. Sci. 2004, 66, 195–210. 10.1007/s00027-004-0710-0.

[ref43] Darnley A. G. Geochemical Atlas of Europe Part 1- Background Information, Methodology and Maps Foreword 2005, No. January 2005.

[ref44] Lenz R.; Enders K.; Nielsen T. G. Microplastic Exposure Studies Should Be Environmentally Realistic. Proc. Natl. Acad. Sci. U. S. A. 2016, 113, E4121–E4122. 10.1073/pnas.1606615113. 27407153 PMC4961204

[ref45] Edgar R. C. UPARSE: Highly Accurate OTU Sequences from Microbial Amplicon Reads. Nat. Methods 2013, 10, 996–998. 10.1038/nmeth.2604. 23955772

[ref46] Wang Q.; Garrity G. M.; Tiedje J. M.; Cole J. R. Naïve Bayesian Classifier for Rapid Assignment of RRNA Sequences into the New Bacterial Taxonomy. Appl. Environ. Microbiol. 2007, 73, 5261–5267. 10.1128/AEM.00062-07. 17586664 PMC1950982

[ref47] Hammer Ø.; Harper D. A. T.; Ryan P. D. Past: Paleontological Statistics Software Package for Education and Data Analysis. Palaeontologia Electronica 2001, 4, 9.

[ref48] Abdolahpur Monikh F.; Chupani L.; Vijver M. G.; Peijnenburg W. J. G. M. Parental and Trophic Transfer of Nanoscale Plastic Debris in an Assembled Aquatic Food Chain as a Function of Particle Size. Environ. Pollut. 2021, 269, 116066 10.1016/j.envpol.2020.116066. 33290950

[ref49] Vardyi S. R.; Warner B. G.; Turunen J.; Aravena R. Carbon Accumulation in Permafrost Peatlands in the Northwest Territories and Nunavut, Canada. Holocene 2000, 10, 273 10.1191/095968300671749538.

[ref50] Yang X.; Jiang C.; Hsu-Kim H.; Badireddy A. R.; Dykstra M.; Wiesner M.; Hinton D. E.; Meyer J. N. Silver Nanoparticle Behavior, Uptake, and Toxicity in *Caenorhabditis Elegans*: Effects of Natural Organic Matter. Environ. Sci. Technol. 2014, 48, 3486–3495. 10.1021/es404444n. 24568198

[ref51] Abdolahpur Monikh F.; Chupani L.; Guo Z.; Zhang P.; Darbha G. K.; Vijver M. G.; Valsami-Jones E.; Peijnenburg W. J. G. M. The Stochastic Association of Nanoparticles with Algae at the Cellular Level: Effects of NOM, Particle Size and Particle Shape. Ecotoxicol. Environ. Saf. 2021, 218, 112280 10.1016/j.ecoenv.2021.112280. 33962275

[ref52] Peng C.; Zhang H.; Fang H.; Xu C.; Huang H.; Wang Y.; Sun L.; Yuan X.; Chen Y.; Shi J. Natural Organic Matter-Induced Alleviation of the Phytotoxicity to Rice (Oryza Sativa L.) Caused by Copper Oxide Nanoparticles. Environ. Toxicol. Chem. 2015, 34, 1996–2003. 10.1002/etc.3016. 25868010

[ref53] Wang X.; Liang D.; Wang Y.; Peijnenburg W. J. G. M.; Monikh F. A.; Zhao X.; Dong Z.; Fan W. A Critical Review on the Biological Impact of Natural Organic Matter on Nanomaterials in the Aquatic Environment. Carbon Res. 2022, 1, 13 10.1007/s44246-022-00013-5.

[ref54] Zhang Z.; Zheng M.; Chen B.; Pan Y.; Yang Z.; Qian H. Nano-Sized Polystyrene at 1 Mg/L Concentrations Does Not Show Strong Disturbance on the Freshwater Microbial Community. Bull. Environ. Contam. Toxicol. 2021, 107, 610–615. 10.1007/s00128-020-02956-0. 32737512

[ref55] Tian W.; Wang H.; Xiang X.; Wang R.; Xu Y. Structural Variations of Bacterial Community Driven by Sphagnum Microhabitat Differentiation in a Subalpine Peatland. Front. Microbiol. 2019, 10, 10 10.3389/fmicb.2019.01661. 31396183 PMC6667737

[ref56] Shi Y.; Li Y.; Xiang X.; Sun R.; Yang T.; He D.; Zhang K.; Ni Y.; Zhu Y.-G.; Adams J. M.; Chu H. Spatial Scale Affects the Relative Role of Stochasticity versus Determinism in Soil Bacterial Communities in Wheat Fields across the North China Plain. Microbiome 2018, 6, 27 10.1186/s40168-018-0409-4. 29402331 PMC5799910

[ref57] Fei Y.; Huang S.; Zhang H.; Tong Y.; Wen D.; Xia X.; Wang H.; Luo Y.; Barceló D. Response of Soil Enzyme Activities and Bacterial Communities to the Accumulation of Microplastics in an Acid Cropped Soil. Sci. Total Environ. 2020, 707, 707 10.1016/j.scitotenv.2019.135634. 31761364

[ref58] Yan Y.; Chen Z.; Zhu F.; Zhu C.; Wang C.; Gu C. Effect of Polyvinyl Chloride Microplastics on Bacterial Community and Nutrient Status in Two Agricultural Soils. Bull. Environ. Contam. Toxicol. 2021, 107, 602–609. 10.1007/s00128-020-02900-2. 32556686

[ref59] Zhu B.-K.; Fang Y.-M.; Zhu D.; Christie P.; Ke X.; Zhu Y.-G. Exposure to Nanoplastics Disturbs the Gut Microbiome in the Soil Oligochaete Enchytraeus Crypticus. Environ. Pollut. 2018, 239, 408–415. 10.1016/j.envpol.2018.04.017. 29679938

