# Supplementary material for: Submicron Plastic Adsorption by Peat, Accumulation in Sphagnum Mosses and Influence on Bacterial Communities in Peatland Ecosystems
Source: Environ Sci Technol. 2022 Nov 3;56(22):15661–71. doi: 10.1021/acs.est.2c04892 (PMC9670847; doi:10.1021/acs.est.2c04892)
Supplement: Supplementary file 1 — es2c04892_si_001.pdf [file es2c04892_si_001.pdf]

Supporting Information

Sub-micron plastic adsorption by peat, accumulation in Sphagnum mosses and influence on Bacterial Communities in Peatland Ecosystems

*Mandar Bandekar [@], Fazel Abdolahpur Monikh [@, ¥, *], Jukka Kekäläinen [@], Teemu Tahvanainen [@], Raine Kortet [@], Peng Zhang [§], Zhiling Guo[§], Jarkko Akkanen [@], Jari T.T. Leskinen [®], Miguel A. Gomez-Gonzalez [ƛ], Gopala Krishna Darbha [µ], Hans-Peter Grossart[¥] , Eugenia Valsami-Jones[§], Jussi V.K. Kukkonen [@].*

[@] Department of Environmental and Biological Sciences, University of Eastern Finland, Joensuu-Kuopio, Finland.

[¥] Department of Experimental Limnology, Leibniz Institute for Freshwater Ecology and Inland Fisheries, Müggelseedamm 310, 12587 Berlin, Germany

[§] School of Geography, Earth and Environmental Sciences, University of Birmingham, Edgbaston, Birmingham, UK.

[®] SIB Labs, University of Eastern Finland, 70211 Kuopio, Finland

[ƛ] Diamond Light Source, Harwell Science and Innovation Campus, Didcot, Oxfordshire OX11 0DE, United Kingdom

[µ] Environmental Nanoscience Laboratory, Department of Earth Sciences, Indian Institute of Science Education and Research Kolkata, Mohanpur, West Bengal, India- 741246.

Section 1. Raman spectroscopy

Raman spectroscopy was used to identify the polymer compositions. The dispersion of 5g L^{-1} of PS-SMPs and the PVC-SMPs were dried on microscope slides for 24 h in darkness at room temperature following the method reported previously [1]. Raman spectra were measured with a Thermo DXR2xi Raman microscope (Thermo Fischer Scientific). The laser wavelength was 785 nm, and the laser power was 30 mW. Measurements were performed for all samples using a grating with 400 lines/mm producing spectral resolution of 5 cm−1 and spectral range of 3300-50 cm−1, exposure time 0.33 s, and the number of scans 40. The polymer compositions of the SMPs were verified by comparing sample spectra to library spectra of plastic polymers.

Section 2. X-ray fluorescence (XRF) microscopy

X-ray fluorescence (XRF) microscopy analyses were carried out on the Hard X-ray Nanoprobe at the I14 beamline (Diamond Light Source). X-rays from an undulator source are pre-shaped using a pair of horizontal mirrors onto a secondary source, with a Si(111) horizontally deflecting double-crystal monochromator providing monochromatic X-rays, selectable over the energy range 5− 23 keV. The beam propagates to an external building (source−sample distance 186 m) where it is focused using pre-shaped KB mirrors. The focused X-ray beam size for this experiment was ~60 nm. The sample is raster scanned (continuous scanning) through the X-ray focus, and the fluorescent X-rays are collected by a four-element silicon drift detector (Rayspec) located in backscatter geometry, with a solid collection angle of 0.8 sr.

A Merlin Quad (Quantum Detectors, UK) photon-counting detector is mounted in transmission geometry for differential phase contrast (DPC) and ptychography imaging (effective pixel <55 microns), which was placed 1.85 meters distant from the sample with a He-filled flight tube in between closing the air gap. The intensity on the detector at this distance can be contained in a single quadrant and is typically

cropped to 128x128 pixels around the nominal beam centre. The masking of the beam is automated to account for small adjustments of the KB mirrors or movement of the Merlin detector.

An Otsu thresholding method can differentiate the beam intensity from the weaker scattering or diffraction signal and is used to identify the region of the beam for the DPC. The phase integration step requires both continuity and differentiability of the phase gradient, which is retrieved by applying a series of fast Fourier Transform and discrete cosine transforms mathematical calculations.

The ptychography scan for the PS-SMPs was acquired out of focus using a 15 ms dwell time over 100 nm intervals. PtyPy was used for data reconstruction [2], which offers ptychographic reconstruction algorithms. The reconstructions presented in this article were carried out as follows: A few hundred (500–1000) iterations of the difference map algorithm were followed by several hundred (1500–3000) iterations of maximum-likelihood refinement, using two mixed states to account for various sources of loss of coherence, yielding the sample's complex-valued transmission function, from which absorption and phase-contrast images can be generated.

Section 3. NOM Preparation

The NOM (1 g) was prepared suspended in MQ water (100 ml) and the pH was adjusted to 8.5 using NaOH (0.01 M). After 24 h of mixing at dark and room temperature using a magnet stirring, the suspension was filtered through a 0.45 μm cellulose acetate membrane and stored at 4 °C until use.

Section 4. Sorption of SMPs to the peat surfaces

The sorption experiments were performed in mesocosms containing peat (without alive mosses) in jars of 200 ml Volume. The jars were put in incubators (MICRO CLIMA-Series™, Snijders Labs Tilburg, the Netherlands) at 20 °C, 16 h / 8 h light:dark cycle and 70% relative humidity. The jars were filled with distilled water and every 48 h the evaporated water was replaced with distilled water to fill the jars again.

Dispersion of approximately 10 mg L^{-1} of SP-SMPs, PVC-SMPs, PS-SMP-NOM or PVC-SMP-NOM were added to the system. Three replicates were used for each treatment. The samples were gently mixed every day using a 10 mL pipette to minimize particle sedimentation. On days 1, 4, 8, 12, and 14, 1 mL of the water samples were taken from the top of the jars after mixing the water and then replaced with 1 mL of distilled water. The samples were measured using spICP-MS to measure the number of the particles and the released Gd ions.

Figure S1 shows the ICP-MS results obtained for Gd in the mesocosms over time. The results showed that the mass concentration of the PS-SMPs and PVC-SMPs decreases over time. This suggests that the particles were removed for the dispersion phase and adsorbed to the surfaces of the peat. In the presence of NOM, however, the particles were stable as that was confirmed by measuring a stable trend for the mass of Gd over time in the dispersion.

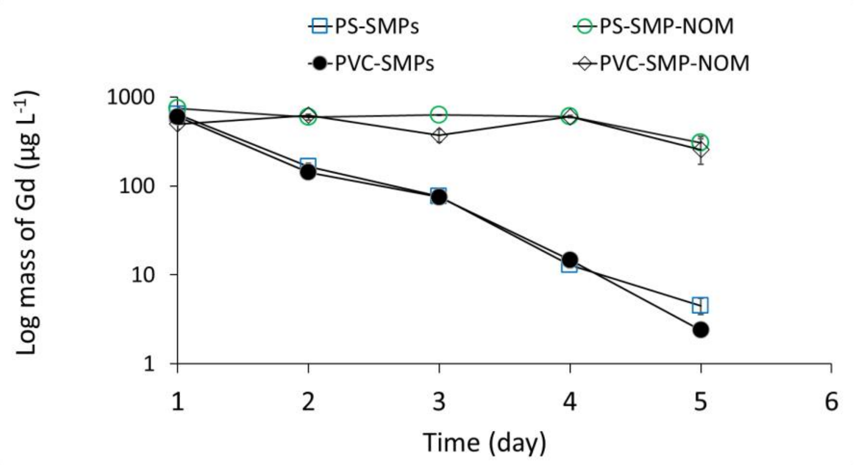

Figure S1. Sorption of SMPs to the peat surface over time. The mass of Gd in the water was measured on days 1, 4, 8, 12, and 14 using ICP-MS.

We tested whether the particles attach to the surface of the jars by performing the experiment without peat under the same conditions. The results showed that there is no significant difference between the number of particles on day 1 and 14 (Figure S2).

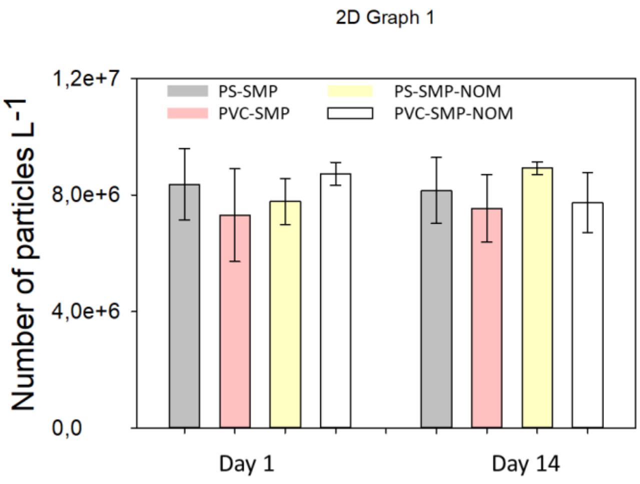

Figure S2. The number of particles on days 1 and 14 in the jars without peats. The results showed that there are no significant differences between the number of the particles on days 1 and 14 [*t-test*, $p < 0.05$, degrees of freedom (df): 2]

Section 5. ICP-MS and spICP-MS measurement

For particle measurement using spICP-MS, the particles first were extracted from the plant tissues using a previously published method [3]. Accordingly, the moss tissues (shoot and leaves) were digested using 10% Tetramethylammonium hydroxide (TMAH). 1 g of plant tissues were put in glass vials and 5 mL of TMAH (10%) was added to the samples. The samples were digested in a water bath at 70 °C in a fume hood until the volume decreased to almost 1 mL. One mL of MQ water was added and the samples were further digested with 30% H_2O_2 for 1 h [4]. After digestion, the samples were diluted with MQ water to reach a final volume of 10 mL and immediately measured using spICP-MS. We have previously tested

that the extraction method (TMAH 5-10% followed by H_2O_2) does not influence the SMPs [3,5] (no particle degradation). No Gd could be measured in the supernatants after particle extraction. The setups used for spICP-MS are summarized in Table S1.

Table S1 | Single-particle inductively coupled plasma mass spectrometry (sPICP-MS) settings

**Single-particle ICP-MS parameters**	
Radio frequency power	1600 W
Nebulizer type	Quartz nebulizer for NexIONs 2000
Spray chamber type	Glass cyclonic
Plasma gas flow	18 L/min
Nebulizer gas flow	1.2 L/min
Auxiliary gas flow	1.12 L/min
Dwell time	80 µs
Acquisition time	100 s

The total mass concertation of the Gd in the samples was measured using ICP-SM. Accordingly, the samples were digested using 2 mL of aqua regia (HNO_3 + 3 HCl) in a water bath at 70 ^{o}C for 1 h. After 1 h, the samples were diluted with 10 mL of MQ water. The samples were further diluted with a dilution factor of 100 with MQ water. The concentration of the Gd in the medium was detected using ICP-MS. The ICP-MS set up summarized in Table S2.

Table S4 | ICP-MS operating conditions

**Parameter/Component**	**Value/Description**
Nebulizer	MEINHARD HEN
Nebulizer gas flow	1.1 L/min
Spray Chamber	Glass cyclonic
RF Power	1600 W

Section 6. Bacterial identification

- Collection of root samples:

Root samples were collected in triplicate for each treatment i.e., CON (3), PS (3), PSNOM (3), PVC (3), and PVCNOM (3). Sampling was done using a clean spatula to remove intact roots from the soil. Samples were placed in sterile bags and immediately stored at -80 C, until further analyses.

- Separation of rhizosphere:

To separate the rhizosphere, 500 mg of each root sample was collected using sterile scissors into a 50 ml conical vial (Tube A) containing 25 ml of epiphyte removal buffer (6.75 g of KH2PO4, 8.75 g of K2HPO4, and 1 mL of Triton X-100, to 1 L of sterile water). The samples were sonicated at 4 °C for 10 min with pulses of 160 W for 30 s. The roots were then transferred to a separate tube (Tube B) using sterile forceps and the original tube (Tube A) containing the buffer and rhizosphere fraction was retained. Tube A was centrifuged (10 min at 4 °C, 4000 x g), the supernatant discarded, and stored at -80°C until DNA extraction.

- DNA extraction:

The sterile spatula was used to transfer 250 mg of rhizosphere sample from each replicate of each treatment into a separate collection tube provided by the commercial DNA isolation kit. DNA extraction was performed following the kit method from ZymoBIOMICS DNA miniprep kit (Zymo Research, USA). The quantity and purity of DNA extracts were determined using Qubit (ThermoFisher Scientific, USA) and Nanodrop (ThermoFisher Scientific, USA).

Section 7. Uptake of NOM-coated SMPs by the shoots

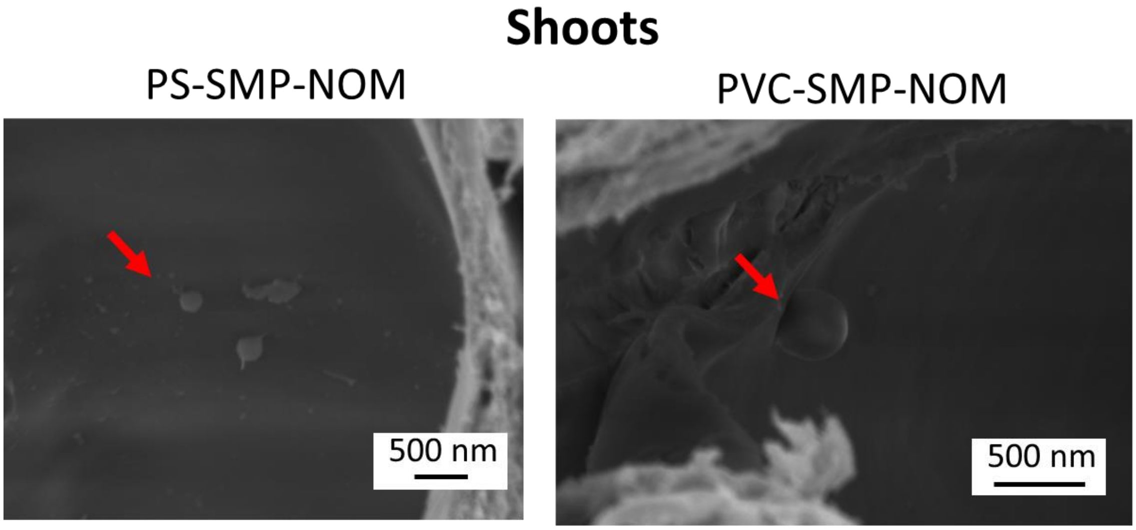

Figure S3. SEM images of PS-SMP-NOM and PVC-SMP-NOM in the shoot of the moss after 14 days of exposure. The red arrows indicate the location of the particles.

Section 8. Presence of SMPs on the external surface of the leaves

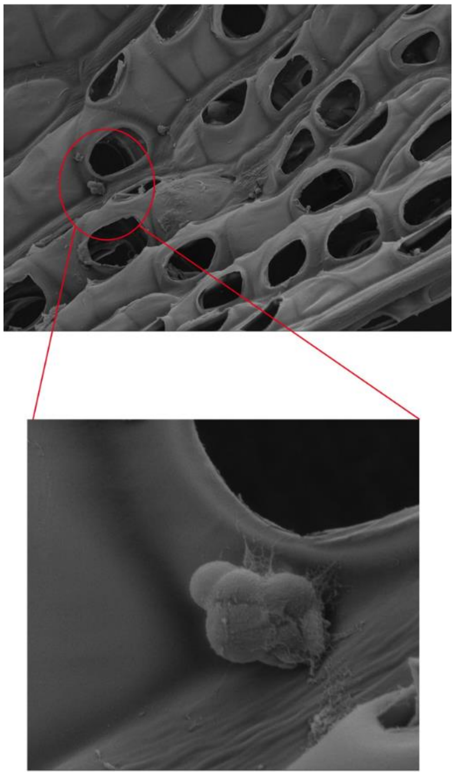

Figure S4. SEM image of PVC-SMPs observed on the external surface of the *Sphagnum* moss leaves.

The effects of SMPs on the microbiome diversity

The saturation of rarefaction curves indicated that bacterial communities were sufficiently deep sequenced (Figure S5).

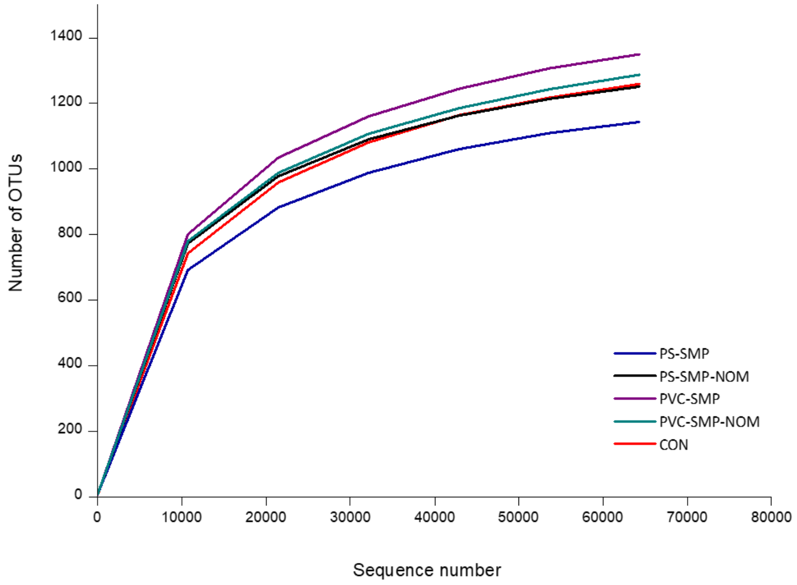

Figure S5. Saturation of rarefaction curves of PS-SMPs, PS-SMP-NOM, PVC-SMPs, PVC-SMP-NOM and CON indicates complete sequencing of all samples.

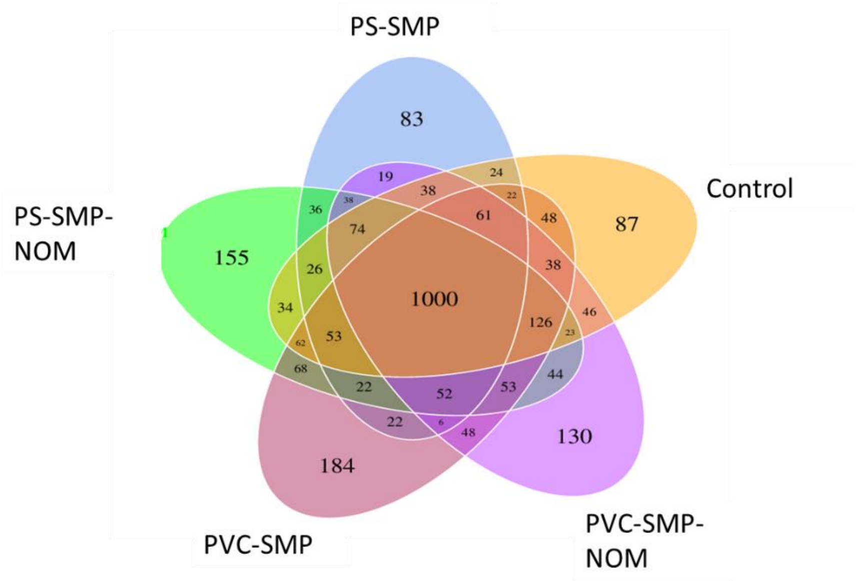

Figure S6. Venn diagram indicating common and unique OTUs at 97% identity among Control (with NOM but no SMPs), PS-SMPs, PS-SMP-NOM, PVC-SMP and PVC-SMP-NOM treatments.

The phylum-level relative abundance was further investigated at the family level (Figures S6).

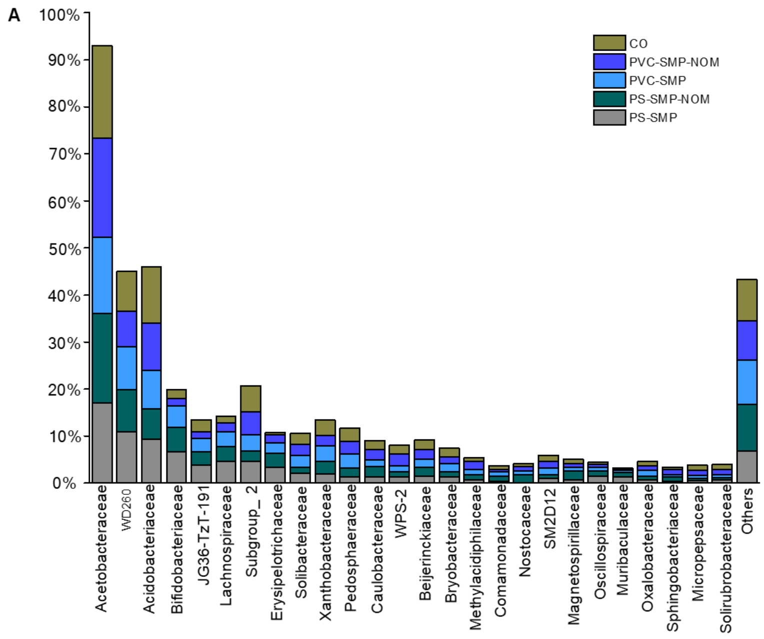

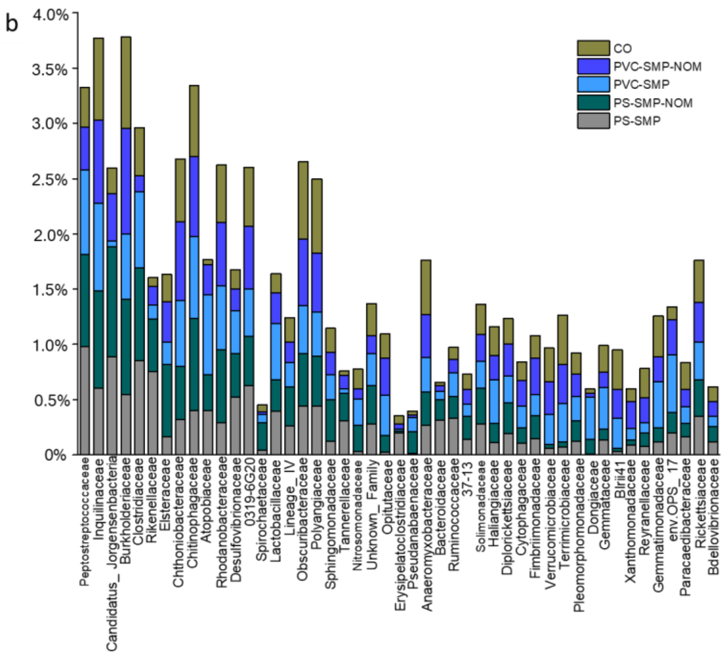

Figure S7. Percentages of relative abundances of major (a) and minor (b) taxa of bacterial communities (at the phylum level) from each PS-SMP, PS-SMP-NOM, PVC-SMP, PVC-SMP-NOM and control without particles. Values represent the means of 3 replicates for each treatment.

References

(1) Monikh, F. A.; Durão, M.; Kipriianov, P. V.; Huuskonen, H.; Kekäläinen, J.; Uusi-Heikkilä, S.; Uurasjärvi, E.; Akkanen, J.; Kortet, R. Chemical Composition and Particle Size Influence the Toxicity of Nanoscale Plastic Debris and Their Co-Occurring Benzo(α)Pyrene in the Model Aquatic Organisms Daphnia Magna and Danio Rerio. *NanoImpact* **2022**, *25*, 100382. https://doi.org/10.1016/j.impact.2022.100382.

(2) Enders, B.; Thibault, P. A Computational Framework for Ptychographic Reconstructions. In *Proceedings of the Royal Society A: Mathematical, Physical and Engineering Sciences*; 2016; Vol. 472, p 20160640. https://doi.org/10.1098/rspa.2016.0640.

(3) Abdolahpur Monikh, F.; Chupani, L.; Vijver, M. G.; Peijnenburg, W. J. G. M. Parental and Trophic Transfer of Nanoscale Plastic Debris in an Assembled Aquatic Food Chain as a Function of Particle Size. *Environmental Pollution* **2021**, *269*, 116066. https://doi.org/10.1016/j.envpol.2020.116066.

(4) Abdolahpur Monikh, F.; Vijver, M. G.; Mitrano, D. M.; Leslie, H. A.; Guo, Z.; Zhang, P.; Lynch, I.; Valsami-Jones, E.; Peijnenburg, W. J. G. M. The Analytical Quest for Sub-Micron Plastics in Biological Matrices. *Nano Today* **2021**, *41*, 101296. https://doi.org/10.1016/j.nantod.2021.101296.

(5) Abdolahpur Monikh, F.; Doornhein, N.; Romeijn, S.; Vijver, M. G.; Peijnenburg, W. J. G. M. Method for Extraction of Nanoscale Plastic Debris from Soil. *Analytical Methods* **2021**, *13* (13),

1576–1583. https://doi.org/10.1039/D0AY02308F.
